# Germplasm Resources Evaluation of Cultured Largemouth Bass (*Micropterus salmoides*) in China Based on Whole Genome Resequencing

**DOI:** 10.3390/genes15101307

**Published:** 2024-10-10

**Authors:** Wenzhi Guan, Jieliang Jian, Baolong Niu, Xinhui Zhang, Jiongying Yu, Xiaojun Xu

**Affiliations:** 1 Institute of Hydrobiology, Zhejiang Academy of Agricultural Sciences, Hangzhou 310021, China; gwzguan@163.com (W.G.); jianjieliang@zaas.ac.cn (J.J.); niubaolong@126.com (B.N.); 2 College of Life Sciences and Oceanography, Shenzhen University, Shenzhen 518060, China; zhangxinhui@genomics.cn; 3 Shenzhen Key Lab of Marine Genomics, Guangdong Provincial Key Lab of Molecular Breeding in Marine Economic Animals, BGI Academy of Marine Sciences, BGI Marine, Shenzhen 518081, China; 4 School of Life Sciences, Huzhou University, Huzhou 313000, China

**Keywords:** largemouth bass, SNP, genetic diversity, population structure, gene flow

## Abstract

**Background:** Largemouth bass (*Micropterus salmoides*), a valuable freshwater fish species, has experienced significant genetic decline in China due to prolonged domestic breeding and limited introduction of new genetic material. It is necessary to have a comprehensive understanding of the genetic status of largemouth bass populations in China. **Method:** In this study, we conducted population genetic analyses on nine cultured largemouth bass populations using whole genome resequencing. **Results:** A total of 3.23 Tb of clean bases were generated, with average Q20 and Q30 values of 98.17% and 94.25%, respectively, and 2,140,534 high-quality SNPs were obtained. Relatively high genetic diversity was observed across all populations. Combined with linkage disequilibrium (LD) patterns, the Wanlu (WL) population possessed the highest genetic diversity, and the Longyou (LY) population possessed the lowest genetic diversity. Additionally, population structure analyses, including pairwise F-statistics, phylogenetic trees, PCA, and admixture analysis, revealed significant genetic differentiation, particularly between the WL, LY, and other 7 populations, while also indicating the occurrence of a common admixture event. Finally, TreeMix inferred migration events from the WL to the Chuanlu (CL) population and from the Taiwan breeding population (TWL) to the Guanglu (GL) population. **Conclusions:** These findings provide a critical foundation for developing conservation and breeding strategies for largemouth bass in China.

## 1. Introduction

Largemouth bass (*Micropterus salmoides*), taxonomically classified in Perciformes, Centrarchidae, and Micropterus, is a freshwater carnivorous fish species native to the Southeastern United States, Northeastern Mexico and Southeastern Canada [1]. Since the 19th century, it has been introduced to numerous countries across Europe [2], Africa [3], and Asia [4], due to its excellent economic traits, such as fast growth, low-temperature resistance, and pleasant flavor without intermuscular bones [5]. Based on the original distribution and morphological characteristics, two subspecies are recognized in largemouth bass: the Northern largemouth bass (*M. salmoides salmoides*) and the Florida largemouth bass (*M. salmoides floridanus*) [6]. In 1983, the Northern largemouth bass was introduced to mainland China from Taiwan, followed by large-scale aquaculture in some Southern provinces such as Guangdong, Zhejiang, Jiangsu, Jiangxi, and Fujian, driven by advancements in artificial compound fodder and food habit domestication [5,7]. By 2023, annual production has reached 888,030 tons, making largemouth bass the seventh most-farmed fish species in China [8]. In addition, largemouth bass has become a focal point for Chinese researchers. In the early stages, around the 1990s, research primarily concentrated on farming technologies. Subsequently, studies expanded to cover areas such as efficient compound feeds, traditional selective breeding, disease prevention and control, and functional analysis of key genes. In recent years, with the advancement of research technologies, fundamental studies on growth, disease, and nutrition, as well as genetics and genomics have flourished comprehensively. However, a series of issues have emerged in the aquaculture of largemouth bass in recent years, primarily manifesting as early sexual maturity, low feed conversion efficiency, reduced growth rate, significant decline in stress resistance, and frequent disease outbreaks [9,10].

High-quality germplasm resources are crucial for the healthy development of the largemouth bass industry. The breeding of largemouth bass began in the 1970s in the United States, focusing on hybrid breeding using two subspecies, while there was no consensus regarding the growth performance of the hybrid offspring [11,12,13]. However, several hybridization attempts conducted in China, particularly those involving native populations and domestic stocks, have demonstrated significant heterosis in growth traits [14,15]. Additionally, triploid largemouth bass were produced through polyploid breeding, preventing genetic introgression during the stocking process [16]. In China, varietal improvement has primarily been achieved through selective breeding, successively developing four largemouth bass varieties or strains: “Youlu No. 1” [17], “Youlu No. 3” [18], “Wanlu No. 1” [19] and “Jia Defeng No. 1” [20], which demonstrate significant advantages in growth characteristics and the domestication success rate. However, despite these advances, the limited renewal of genetic resources, the prolonged domestic culture, the short breeding cycles, as well as the chaotic and disorganized exchange of breeding populations, led to the decline of germplasm resources.

To design effective breeding programs, a comprehensive investigation into the genetic background of largemouth bass populations in China is requisite. Historical studies carried out thus far usually focused on the genetic diversity analysis among native populations, domestic farmed populations, and hybrid populations using random amplified polymorphic DNA (RAPD) [21,22], mitochondrial DNA [23,24], or microsatellites [1,4,25]. These studies collectively depicted the following two conditions regarding largemouth bass in China: the genetic diversity of domestically farmed populations is lower than that of native populations, and there is evidence of genetic admixture among domestic farmed populations. While a thorough understanding remains elusive due to the limited molecular markers and sample size. With the rapid development of high-throughput sequencing technologies, single nucleotide polymorphism (SNP)-based genotyping is revolutionizing the genetics field, and has been a powerful tool in population genetic analysis, enabling more precise and comprehensive analyses by providing a detailed, genome-wide view of genetic variation. In previous studies, Du et al. [26] utilized resequencing data from ten individuals of the Northern largemouth bass and ten individuals of the Florida largemouth bass to explore their genetic diversity and discovered that the germplasm quality of the Northern largemouth bass had markedly diminished in China. Additionally, Sun et al. [27] assessed genome-wide diversity and identified selective signatures using whole-genome resequencing data from five populations, and found a decline in genetic diversity within the domestic breeding populations. The aforementioned results unequivocally indicated that an investigation and improvement of the genetic resources of domestic largemouth bass populations is of great necessity. In the present study, we conducted a population genetic analysis on nine largemouth populations, including five newly sampled populations and four populations previously sequenced by Sun et al. [27], using whole-genome sequencing data. Analyses of genetic diversity, population differentiation, and gene flow were carried out, aiming to provide a comprehensive understanding of the genetic background of largemouth bass populations in China and enhance scientific management and breeding strategies.

## 2. Materials and Methods

### 2.1. Sample Collection

A total of 169 individuals from five largemouth bass populations were sampled. Specifically, the Bushen population (BS, *n* = 24) and the Longyou population (LY, *n* = 35) were sampled from the Bolongkeng reservoir and Bashiban reservoir in Quzhou, Zhejiang, China, respectively, which have undergone long-term self-breeding since the 1990s. The Taiwan breeding population (TWL, *n* = 30) was obtained from Zhangzhou Qunfu Biotechnology Co., Ltd. (Zhangzhou, Fujian, China), and the common farming populations, Chuanlu (CL, *n* = 50) and Guanglu (GL, *n* = 30), were provided by Panzhihua Laibei Aquatic Co., Ltd. (Panzhihua, Sichuan, China) and Tongtian Aquatic Co., Ltd. (Foshan, Guangdong, China), respectively. The caudal fin of each individual was collected and preserved in 95% ethanol and stored at −20 °C.

### 2.2. DNA Extraction and Sequencing 

The genomic DNA of each sample was extracted using a QIAamp DNA Mini Kit (Qiagen, Hilden, Germany) according to the manufacturer’s instructions. The integrity and purity of DNA were determined via 1% agarose gel electrophoresis and an Agilent 4200 Bioanalyzer (Agilent Technologies, Santa Clara, CA, USA), respectively, and the DNA concentration was evaluated using a Qubit 2.0 fluorometer (Life Technologies, Gaithersburg, MD, USA). High-quality DNA was used to construct paired-end DNA libraries with an insert size of 350 bp using a Genomic DNA Sample Prep Kit (Illumina, San Diego, CA, USA), and then the qualified libraries were sequenced on the DNBSEQ-T7 platform (BGI Tech, Shenzhen, China) in paired-end mode (2 × 150 bp).

### 2.3. Genotyping

Sequencing data obtained in our study, together with the downloaded data of one US-introduced protospecies (WL, *n* = 29, obtained from Auhui Zhanglin Fishery Co., Ltd., Tongling, China) and three breeding populations, namely Youlu breeding population (YL, *n* = 30, obtained from Aiyu breeding Co., Ltd., Guangdong, China), Jiadefeng breeding population (JDF, *n* = 30, obtained from Foshan Xinrong Aquatic Co., Ltd., Foshan, Guangdong, China), and common breeding population in Guangdong (TL, *n* = 30, obtained from Foshan Xinrong Aquatic Co., Ltd., Guangdong, China) [27], were utilized to perform genotyping and the subsequent genetic analyses. Initially, raw data were filtered using SOAPnuke v1.5.6 [28] software to remove sequences containing primers or adapters, low-quality sequences with base quality value ≤ 20, and sequences with an N ratio exceeding 5%. Then the clean data were aligned to the reference genome of largemouth bass (NCBI No. ASM1485139) using the “mem” algorithm in BWA v0.7.15 software with default parameters [29]. The obtained SAM format files were then converted to BAM format using SAMtools v1.9 software [30], and PCR duplicates were removed using MarkDuplicates in Picard Tools v2.13.2 (http://broadinstitute.github.io/picard/) (accessed on 25 September 2024). SNP calling was carried out using the Genome Analysis Toolkit (GATK) v4.0 [31] with default settings. To ensure the reliability of the subsequent analysis, the obtained SNPs were filtered using GATK VariantFiltration with the following parameters: SNPs with QD < 2.0, FS > 60.0, MQ < 40.0, MQRankSum < −12.5, and ReadPosRankSum < −8.0. Then each SNP was further filtered at the population level using VCFtools v0.1.13 [32] if: (1) minor allele frequency < 0.05, (2) samples with missing genotypes > 0.1, and (3) sequencing depth < 4. Finally, PLINK v1.9 [33] was applied to perform linkage disequilibrium pruning with a window size of 10 kb, step size of one SNP, and r^2^ of 0.5, avoiding the influence of highly correlated SNPs on population structure analysis.

### 2.4. Genetic Diversity and Linkage Disequilibrium Analysis

Genetic parameters, including observed heterozygosity (*HO*), expected heterozygosity (*HE*), and nucleotide diversity (*Pi*), were analyzed using VCFtools v0.1.13. *Pi* was analyzed using a sliding-window approach with a window size of 5 kb. The genome inbreeding coefficients (*Froh*), defined as the sum of ROH (runs of homozygosity) lengths of an individual divided by the total length of the autosomes, were calculated using PLINK v1.9 with the following comments: --homozyg-window-snp 50 --homozyg-density 50 --homozyg-window-het 3 --homozyg-window-missing 1. Additionally, PopLDdecay v3.40 [34] was used to perform linkage disequilibrium (LD) analysis among all populations, and LD plots were drawn using the Plot_MultiPop module.

### 2.5. Population Structure Analysis

To understand the genetic structure of the investigated populations, a phylogenetic tree was constructed using the neighbor-joining (NJ) method in PHYLIPNEW v3.69 (https://emboss.sourceforge.net/apps/cvs/embassy/phylipnew/) (accessed on 25 September 2024), incorporating 100 bootstraps, and the tree was visualized with iTOL (https://itol.embl.de) (accessed on 25 September 2024). Principal components analysis (PCA) was performed with FlashPCA v1.2.6 [35], and the results were plotted using the first and second eigenvalues in the “ggplot2” package in R v4.4.0 [36]. Additionally, Admixture v1.3.0 software [37] was utilized to analyze the population structure, with the assumed number of genetic groups (K-value) ranging from 2 to 11. For each K-value, ten independent runs were performed. The K-value yielding the lowest cross-validation error (CV error) was identified as the optimal representation of the population structure. Additionally, pairwise F-statistics (*Fst*) among populations were analyzed using VCFtools (v0.1.13) with a sliding window of 100 kb and a step size of 10 kb. Genetic differentiation level among populations represented by *Fst* was categorized into huge differentiation (*Fst* > 0.25), great differentiation (0.15 < *Fst* < 0.25), moderate differentiation (0.05 < *Fst* < 0.15), and negligible differentiation (*Fst* < 0.05) [38].

### 2.6. Gene Flow Analysis

To investigate genetic drift and infer gene flows resulting from historical population splits and mixtures among the nine largemouth bass populations, we used TreeMix (v 1.13) software [39] to explore the gene flow events based on genome-wide allele frequency data. The number of migration events (m) was postulated to be 0–10, and analysis for each case was repeated five times. The output files generated by TreeMix were further examined using the “OptM” package [40] to identify the optimal m value, with 500 bootstrap replicates. Finally, the tree and residuals plot with the optimal migration events were visualized using the R script “plotting_funcs” embedded in TreeMix.

## 3. Results

### 3.1. Summary of Sequencing and SNP Calling

A total of 22.3 billion 150 bp paired-end raw reads with an average depth of 20.63× were generated through whole-genome resequencing of 169 individuals from five populations. After filtering, a total of 1.91 Tb of clean bases were obtained, with average Q20 and Q30 values of 98.18% and 94.16%, respectively (Table S1). Additionally, 1.35 Tb of clean bases from another four populations were downloaded from the NCBI database [29]. All clean data were aligned to the reference genome for SNP calling, resulting in 6,357,890 SNPs. After further filtering, 2,140,534 high-quality SNPs were retained for subsequent analysis.

### 3.2. Results of Genetic Diversity and LD Analysis

**Figure 1:**
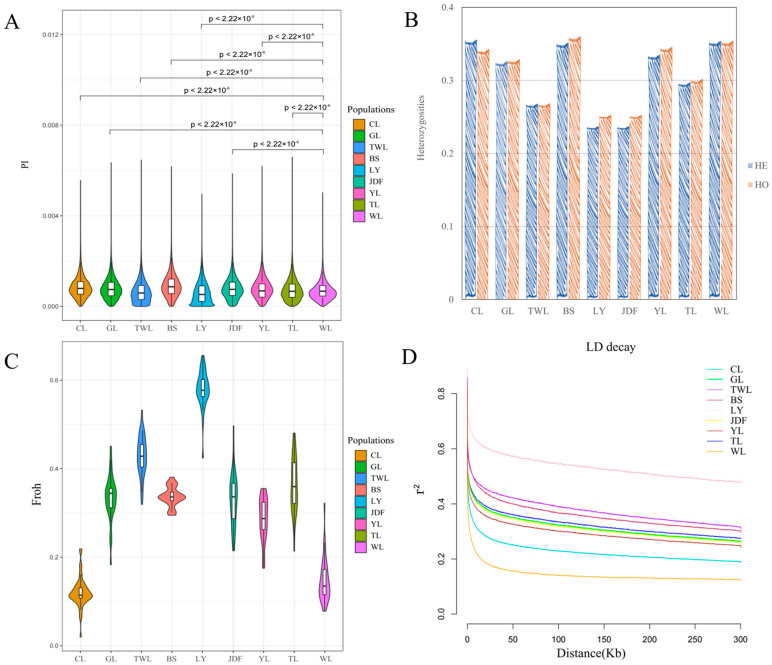
Genetic diversity measurement of the nine investigated largemouth bass populations. (**A**) Violin plot of nucleotide diversity (*Pi*) for each population in 100 kb windows with 10 kb steps. (**B**) Expected and observed heterozygosities of each population. (**C**) Violin plot of genome inbreeding coefficient (Froh) for each population. (**D**) LD decay pattern.

The genetic parameters across the nine largemouth bass populations were analyzed to assess their genetic diversity. Nucleotide diversity (*Pi*) displayed notable variability, ranging from 6.2 × 10^−^^4^ in the LY population to 9.3 × 10^−^^4^ in the WL population, with an overall average of 7.9 × 10^−^^4^. Significant differences emerged between the WL and the remaining eight populations (*p* < 0.05), highlighting the distinct genetic makeup of WL (Figure 1A). The *HE* values varied from 0.2371 to 0.3556 and the *HO* values varied from 0.2525 to 0.3604, with average values of 0.3265 and 0.3204, respectively. The highest *HE* and *HO* values were observed in the CL and BS populations, and the lowest *HE* and *HO* values were observed in the LY population (Figure 1B). Estimates of the genome inbreeding coefficients (Froh) among all populations ranged from 0.1181 (LY) to 0.5797 (WL) (Figure 1C). Collectively, according to the estimates of these four genetic parameters, the genetic diversity of the nine populations was ranked (from high to low) as follows: WL > CL > YL > BS > JDF > GL > TL > TWL > LY.

Elucidating the patterns of LD is crucial for population genetic analysis. The LD decay distances, defined as the point at which LD (r^2^) decayed to half its maximum value, varied considerably: from 557 bp in CL (r^2^ = 0.435) to 243.37 kb in LY (r^2^ = 0.468) (Figure 1D). Overall, the LY population exhibited the slowest LD decay and highest LD level, while the WL population exhibited the fastest LD decay and lowest LD level, suggesting less selective pressure and greater genetic diversity. These findings align with the genetic parameter evaluations, further confirming that the WL population harbors the most genetic diversity, whereas the LY population shows evidence of significant inbreeding and reduced variability. Furthermore, the YL and JDF populations, two formally bred varieties or strains, exhibited faster LD decay rates than those of the common breeding populations (TL, GL), indicating their relatively higher genetic diversity.

### 3.3. Results of Genetic Differentiation

**Figure 2:**
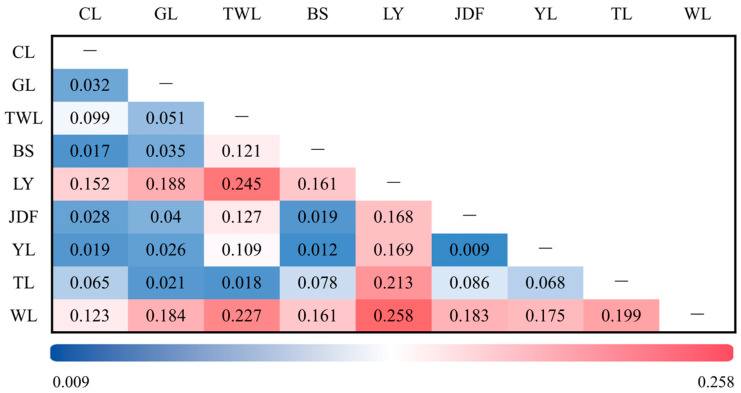
Pairwise *Fst* among the nine investigated largemouth bass populations.

The pairwise F-statistics (*Fst*) were calculated to investigate the genetic differentiation. *Fst* values observed between all populations ranged from 0.009 to 0.258. Huge differentiation with an *Fst* value of 0.258 was merely observed between population LY and WL. High differentiation with *Fst* values ranging from 0.152 to 0.245 was observed between LY, WL, and the remaining seven populations, except for the moderate differentiation between WL and CL. While negligible differentiation or moderate differentiation with *Fst* ranging from 0.009 to 0.127 were observed between populations BS, CL, TL, GL, JDF, TWL and YL (Figure 2).

**Figure 3:**
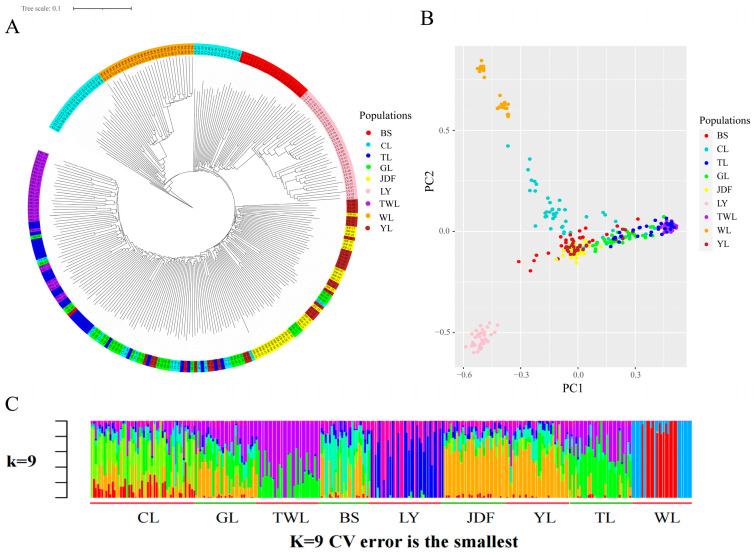
Results of population structure analyses. (**A**) A phylogenetic tree for the 288 largemouth bass individuals. (**B**) A PCA plot with the first and second eigenvectors. (**C**) Population structure when K = 9, where the length of each colored segment represented the proportion of the individual genome inferred from ancestral populations.

To further elucidate the genetic relationships among the nine populations, population structure was analyzed. First, the reconstructed phylogenetic tree clustered the 288 individuals into three clades, with bootstrap values at root node and internal nodes exceeding 70: all individuals from the WL population and 21 individuals from the CL population formed one cluster; all individuals from LY and BS populations, along with another 14 individuals from the CL population, formed the second cluster; all individuals from TL, GL, JDF, YL, and TWL populations, along with the remaining 15 individuals from the CL population, formed the third cluster, with individuals from each population interspersed (Figure 3A). These findings were subsequently supported by the PCA plot, which highlighted a clearer separation of the WL and LY populations from the others, reinforcing their distinct genetic profiles (Figure 3B). Admixture analysis provided additional insight into the ancestral composition of each individual. The cross-validation error value was lowest when K was nine, and the result showed evidence of admixture, with each population displaying multiple ancestral components (Figure 3C).

### 3.4. Gene Flow

**Figure 4:**
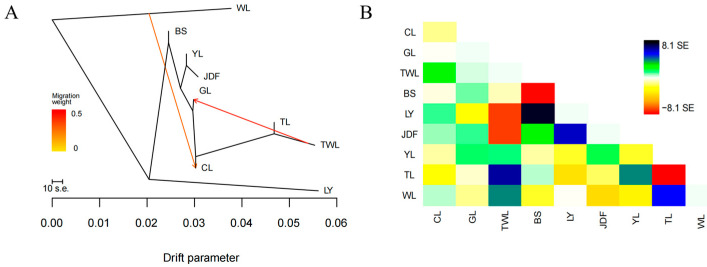
Results of gene flow analysis. (**A**) Maximum likelihood tree. Arrows indicated migration event and the heat map indicated migration weight. (**B**) Scaled residual fit between observed data and predicted model with two migration events.

The events of gene flow among the nine populations were inferred using Treemix software. The optimal number of migration events (m) estimated by the “optM” package was two, suggesting two gene flow events occurred among the investigated populations. The inferred phylogenetic network divided all populations into three main branches. WL and LY each represented a single branch, and the remaining populations clustered into a single branch. Events of gene flow from TWL to GL and from WL to CL were observed (Figure 4).

## 4. Discussion

As an important economic freshwater fish species, largemouth bass has been favored by customers and farmers in the past decades. However, the prolonged domestic culture, limited introduction of new genetic material, and inbreeding have led to a serious germplasm decline in largemouth bass in China [41]. Despite the effective promotion of largemouth bass aquaculture through the development of several new varieties in recent years, such as *M. salmoides* “Youlu No. 1” and *M. salmoides* “Youlu No. 3”, the demand for high-quality germplasm remains ongoing. Thus, to investigate the genetic background of the primary domestic breeding strains and some potential breeding resources, we conducted a comprehensive population genetic analysis using whole-genome sequencing data from nine largemouth bass populations in the present study.

Genetic parameters, including nucleotide diversity, heterozygosity, and inbreeding coefficients were assessed in our study. Nucleotide diversity ranged from 6.2 × 10^−^^4^ to 9.3 × 10^−^^4^ was observed in these nine largemouth bass populations, greatly lower than that observed in large yellow croaker (*Larimichthys crocea*) [42] and black carp (*Mylopharyngodon piceus*) [43], which might be attributed to species-specific factors such as low mutation rate, low recombination rate, and high selection pressure [44]. Expected heterozygosity and observed heterozygosity indicated a broad range of genetic diversity among the studied populations, yet overall remained at a relatively high level compared to many other aquaculture species [43,45,46]. Notably, the genetic diversity of the WL and CL populations was relatively high. The foundational populations of WL and CL were reported to be introduced domestically in 2016, the most recent effort to directly introduce native largemouth bass from the United States [19]. The relatively short history of their domestication and acclimatization explained their high genetic diversity. In contrast, the LY and TWL populations exhibited lower genetic diversity. The LY population has been cultivated in an isolated reservoir for several decades, with a portion of the broodstock retained each year for breeding purposes, and thus we attributed the low genetic diversity to intensive selective breeding practices and inbreeding, further corroborated by the inbreeding coefficient. Similarly, the BS population, which shares the same foundational population as the LY population and has undergone similar breeding strategies in another reservoir, nonetheless exhibited higher genetic diversity, suggesting the possible introduction of external genetic material. The TWL population, introduced from Taiwan, displayed low genetic diversity, likely due to its early introduction from the United States and subsequent multiple rounds of artificial selection, and a similar pattern was also reported by Su et al. [47]. Lastly, the YL and JDF selectively bred populations maintained relatively high genetic diversity, suggesting that effective breeding management and conservation strategies have been implemented in these populations.

Linkage disequilibrium (LD) analysis was performed, and the pattern of LD decay generally supported the above findings. The WL population exhibited the fastest LD decay and the lowest LD levels, indicating a high level of genetic recombination and low selection pressure. In contrast, the LY population showed the slowest LD decay and the highest LD levels. Studies suggested that long-term isolated populations typically exhibit two main trends: (i) low genetic variation and (ii) high linkage disequilibrium [48,49]. However, the BS population, which had relatively high genetic diversity, paradoxically exhibited high linkage disequilibrium and low decay rate. Similar results have also been observed in the Ashkenazi Jewish population, where events of admixture were considered a causal reason [50]. Generally, elevated LD could result from genetic bottlenecks, inbreeding, or selection, while these events do not account for high genetic diversity. However, elevated LD and genetic diversity could simultaneously arise from the admixture of genetically distinct populations [51,52]. Thus, events of admixture were possible in the BS population.

Pairwise *Fst* values, reconstructed phylogenetic, PCA plot, and admixture analysis were used to elucidate the genome-wide genetic structure of these nine populations. Pairwise *Fst* values showed huge differentiation between WL and LY populations and high genetic differentiation between LY and WL populations and the remaining populations. The phylogenetic tree and PCA plot consistently demonstrated the genetic separation of the WL and LY populations, highlighting their distinct genetic backgrounds. Additionally, the structure analysis further indicated that each population experienced events of admixture. Overall, the genetic structure observed here corroborated the historical introduction of largemouth bass into mainland China. For instance, the TL population, which was introduced earliest from Taiwan but underwent events of admixture and interbreeding, showed the lowest genetic differentiation with the TWL population, recently introduced from Taiwan. Then, the WL population, which was the most recently introduced native population, showed high genetic differentiation with most populations except the CL population. Notably, the BS population, which was also cultivated in isolation in a reservoir, showed close genetic relationships with most other populations, which supported the above hypothesis that external germplasm was introduced to the BS population, and the structure analysis indicated that the external germplasm might be YL population. Additionally, the GL population is a common breeding population cultivated in the Guangdong province. Its genetic background has been relatively underexplored, as this population has experienced numerous untraceable and undocumented germplasm introductions. The results of our study confirmed the complex genetic background of GL, with preliminary speculation suggesting that the GL population is primarily composed of a self-propagating population, the Taiwan population, and the Youlu population. Intriguingly, the CL population, apart from certain individuals, was distributed across two distinct phylogenetic branches, and the genetic diversity was second only to that of WL. We speculated that the introduction of external germplasm with low genetic diversity might be the underlying cause. Finally, the WL population exhibited a certain degree of gene flow to the CL population but has not yet undergone significant admixture with other populations, indicating effective and systematic conservation efforts.

## 5. Conclusions

In conclusion, our study provides a comprehensive understanding of the genetic background of cultured largemouth bass populations in China. The results revealed relatively high genetic diversity in these studied largemouth bass populations and indicated frequent events of germplasm exchange. Moreover, the WL and LY populations were suggested to have significant potential for future selection and hybrid breeding programs. Overall, these findings offer valuable insights for developing scientific conservation and breeding strategies. 

## Data Availability

All sequencing data have been uploaded to the National Center for Biotechnology Information (NCBI) SRA (Sequence Read Archive) database under PRJNA1149185.

## Supplementary Materials

The following supporting information can be downloaded at: https://www.mdpi.com/article/10.3390/genes15101307/s1, Table S1: Statistics for the Whole-genome sequencing data.
